# Supplementary material for: Patterns of genetic structuring at the northern limits of the Australian smelt (Retropinna semoni) cryptic species complex
Source: PeerJ. 2018 May 3;6:e4654. doi: 10.7717/peerj.4654 (PMC5936633; doi:10.7717/peerj.4654)
Supplement: Table S1 — Departures from Hardy-Weinberg equilibrium (following Bonferroni correction) are indicated by an asterisk (∗). [file peerj-06-4654-s001.docx]

| Population name | | | | | | | | | | | | | | | |
| --- | --- | --- | --- | --- | --- | --- | --- | --- | --- | --- | --- | --- | --- | --- | --- |
| Locus | MRD | MRU | NSD | MLD | MLU | BRD | BRU | LAD | LAU | CMD | CMU | NRD | NRU | CRD | CRU |
| BS18 | 0.588 | 0.407 | 0.968 | 0.756 | 0.826 | 0.824 | 0.137 | 0.089 | 0.445 | 0.603 | 0.302 | 0.550 | 0.065 | 0.849 | 0.138 |
| BS3 | 0.079 | 0.989 | 0.072 | 0.431 | 0.281 | 0.167 | 0.852 | 0.590 | 0.494 | 0.077 | 0.098 | 0.034 | 0.017 | 0.250 | 0.721 |
| BS4 | 0.076 | 0.676 | 1.000 | 0.704 | 0.230 | 0.982 | 0.273 | 0.485 | 0.884 | 0.955 | 0.259 | 0.552 | 0.346 | 0.393 | 0.892 |
| BS5 | 0.501 | 0.259 | 0.846 | 0.263 | 0.618 | 0.914 | 0.453 | 0.811 | 0.239 | 0.185 | 0.237 | 0.685 | 0.396 | 0.960 | 0.475 |
| BS22 | 0.241 | 0.111 | 0.052 | Monomorphic | 1.000 | 0.124 | 0.631 | 0.224 | 0.824 | 0.040 | 0.329 | 1.000 | 0.004 | 0.187 | 0.636 |
| BS20 | 0.328 | 0.508 | 0.011 | 0.056 | 0.094 | 0.693 | 0.746 | 0.008 | 0.010 | 0.233 | 0.042 | 0.914 | 0.000* | 0.059 | 0.262 |
| BS21 | 0.355 | 0.183 | 0.103 | 0.286 | 0.000* | 0.078 | 0.129 | 0.427 | 0.496 | 0.633 | 0.734 | 0.099 | 0.000* | 0.092 | 0.165 |
| BS24 | 0.006 | 0.011 | 0.057 | 1.000 | 0.012 | 0.681 | 0.112 | 0.173 | 0.314 | 0.615 | 0.110 | 0.360 | 0.587 | 0.091 | 0.077 |
| BS8 | 0.329 | 0.933 | 0.890 | 1.000 | 0.326 | 0.154 | 0.252 | 0.371 | 0.207 | 0.415 | 0.573 | 0.751 | 0.025 | 0.780 | 0.955 |
| MS24 | 0.000* | 0.245 | 0.057 | 0.359 | 0.003* | 0.902 | 0.725 | 0.234 | 0.188 | 0.494 | 0.420 | 0.157 | 0.293 | 0.299 | 0.006 |
