# Supplementary material for: Patterns of genetic structuring at the northern limits of the Australian smelt (Retropinna semoni) cryptic species complex
Source: PeerJ. 2018 May 3;6:e4654. doi: 10.7717/peerj.4654 (PMC5936633; doi:10.7717/peerj.4654)
Supplement: Table S2 — No means no evidence for null alleles. See Table 1A for site codes and locations. [file peerj-06-4654-s002.docx]

| Population | Locas name | | | | | | | | | |
| --- | --- | --- | --- | --- | --- | --- | --- | --- | --- | --- |
|  | BS18 | BS3 | BS4 | BS5 | BS20 | BS21 | BS22 | BS24 | BS8 | MS24 |
| MRD | no | no | no | no | no | no | no | 0.155 | no | 0.144 |
| MRU | no | no | no | no | 0.153 | no | no | 0.137 | no | no |
| NSD | no | no | no | no | no | no | no | no | no | no |
| MLD | no | no | no | no | no | no | no | no | no | no |
| MLU | no | no | no | no | no | no | no | no | no | no |
| BRD | no | no | no | no | no | no | no | no | no | no |
| BRU | no | no | no | no | 0.189 | no | no | no | no | no |
| LAD | no | no | no | no | no | no | no | no | no | no |
| LAU | no | no | no | no | no | no | no | no | no | no |
| CMD | no | no | no | no | no | no | no | no | no | no |
| CMU | no | no | no | no | no | no | no | no | no | no |
| NRD | no | 0.133 | no | no | no | no | no | no | no | no |
| NRU | no | no | no | no | 0.271 | no | 0.097 | no | no | no |
| CRD | no | no | no | no | no | 0.073 | no | no | no | no |
| CRU | no | no | no | no | no | no | no | 0.073 | no | no |
