# Supplementary material for: Patterns of genetic structuring at the northern limits of the Australian smelt (Retropinna semoni) cryptic species complex
Source: PeerJ. 2018 May 3;6:e4654. doi: 10.7717/peerj.4654 (PMC5936633; doi:10.7717/peerj.4654)
Supplement: Table S3 — The mean and standard deviations (SD) of the log-likelihood values (LnP[D]) for different hypothesised numbers of genetic populations (K). The mean value of K, the ad hoc statistic of Evanno, Regnaut & Goudet (2005) was used to summerize the second-order rate of change in LnP(D). The bold value of K = 8 represents the most likely number of genetic groups indicated by both test statistics. NA = not applicable given that K cannot be calculated for these value of K. [file peerj-06-4654-s003.docx]

| K | Mean LnP(D) | SD LnP(D) | ∆*K* |
| --- | --- | --- | --- |
| 1 | -22013.1 | 0.555878 | NA |
| 2 | -19812.4 | 131.081 | 6.543053 |
| 3 | -18469.2 | 325.2155 | 1.31482 |
| 4 | -17553.7 | 128.9415 | 0.229251 |
| 5 | -16608.7 | 130.2262 | 3.075878 |
| 6 | -16064.2 | 75.74812 | 0.278291 |
| 7 | -15540.7 | 114.4082 | 0.945124 |
| 8 | **-15125.4** | 0.584618 | **1741.891** |
| 9 | -15728.5 | 315.4953 | 0.793895 |
| 10 | -16081.0 | 785.5523 | 0.328113 |
| 11 | -16691.4 | 995.1 | 1.089247 |
| 12 | -16217.8 | 449.791 | 2.056355 |
| 13 | -16669.1 | 844.5295 | 0.350562 |
| 14 | -16824.4 | 827.0747 | 0.693081 |
| 15 | -16406.4 | 550.8407 | 1.219772 |
| 16 | -16660.4 | 429.0257 | NA |
