# Supplementary material for: Patterns of genetic structuring at the northern limits of the Australian smelt (Retropinna semoni) cryptic species complex
Source: PeerJ. 2018 May 3;6:e4654. doi: 10.7717/peerj.4654 (PMC5936633; doi:10.7717/peerj.4654)
Supplement: Table S4 [file peerj-06-4654-s004.docx]

| Sample Code | Genbank Accession number |
| --- | --- |
| BDC1 | MG867590 |
| BDC10 | MG867591 |
| BDC2 | MG867592 |
| BDC3 | MG867593 |
| BDC4 | MG867594 |
| BDC5 | MG867595 |
| BDC6 | MG867596 |
| BDC7 | MG867597 |
| BDC8 | MG867598 |
| BDC9 | MG867599 |
| BRD1 | MG867600 |
| BRD2 | MG867601 |
| BRU1 | MG867602 |
| BRU2 | MG867603 |
| BSV1 | MG867604 |
| BSV10 | MG867605 |
| BSV2 | MG867606 |
| BSV3 | MG867607 |
| BSV4 | MG867608 |
| BSV5 | MG867609 |
| BSV6 | MG867610 |
| BSV7 | MG867611 |
| BSV8 | MG867612 |
| BSV9 | MG867613 |
| CMD1 | MG867614 |
| CMD2 | MG867615 |
| CMU1 | MG867616 |
| CMU2 | MG867617 |
| CRD1 | MG867618 |
| CRD2 | MG867619 |
| CRU1 | MG867620 |
| CRU2 | MG867621 |
| LAD1 | MG867622 |
| LAD2 | MG867623 |
| LAU1 | MG867624 |
| LAU2 | MG867625 |
| MBC1 | MG867626 |
| MBC2 | MG867627 |
| MBC3 | MG867628 |
| MBC4 | MG867629 |
| MBC5 | MG867630 |
| MBC6 | MG867631 |
| MBC7 | MG867632 |
| MBC8 | MG867633 |
| MBC9 | MG867634 |
| MLD1 | MG867635 |
| MLD2 | MG867636 |
| MLU1 | MG867637 |
| MLU2 | MG867638 |
| MLU3 | MG867639 |
| MRD1 | MG867640 |
| MRD2 | MG867641 |
| MRU1 | MG867642 |
| MRU2 | MG867643 |
| MYC1 | MG867644 |
| MYC2 | MG867645 |
| MYC3 | MG867646 |
| MYC4 | MG867647 |
| MYC5 | MG867648 |
| MYC6 | MG867649 |
| MYC7 | MG867650 |
| MYC8 | MG867651 |
| NRD1 | MG867652 |
| NRD2 | MG867653 |
| NRU1 | MG867654 |
| NRU2 | MG867655 |
| NSD1 | MG867656 |
| NSD2 | MG867657 |
