# Supplementary figures and images for: Patterns of genetic structuring at the northern limits of the Australian smelt (Retropinna semoni) cryptic species complex
Source: PeerJ. 2018 May 3;6:e4654. doi: 10.7717/peerj.4654 (PMC5936633; doi:10.7717/peerj.4654)

$$\text{DeltaK} = \text{mean}(|L''(K)|) / \text{sd}(L(K))$$

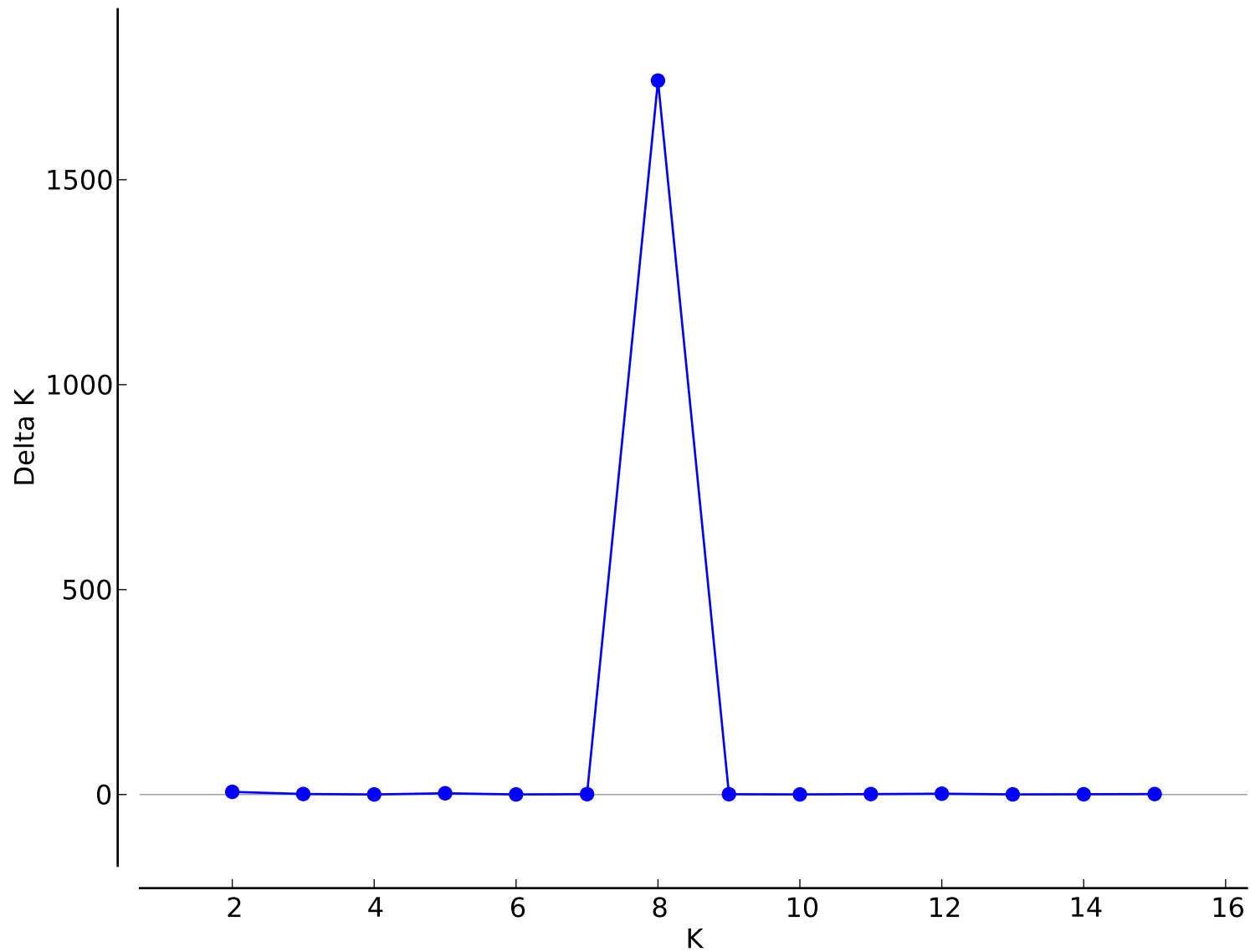

Supplement: Figure S1 — K means number of clusters and ΔK means the corresponding ΔK statistics calculated according to Evanno, Regnaut & Goudet (2005). [file peerj-06-4654-s005.pdf]
